# Preoperative management comprising tube irrigation using a trans-anal indwelling tube for infants with hirschsprung disease can allow single-stage radical surgery

**DOI:** 10.1186/s12893-023-02232-y

**Published:** 2023-11-01

**Authors:** Yoichi Nakagawa, Hiroo Uchida, Akinari Hinoki, Takahisa Tainaka, Chiyoe Shirota, Wataru Sumida, Satoshi Makita, Kazuki Yokota, Hizuru Amano, Akihiro Yasui, Takuya Maeda, Daiki Kato, Yousuke Gohda

**Affiliations:** 1 Department of Pediatric Surgery, Nagoya University Graduate School of Medicine, 65 Tsurumai-cho, Showa-ku, Nagoya, 466-8550 Japan; 2 Department of Rare/Intractable Cancer Analysis Research, Nagoya University Graduate School of Medicine, 65 Tsurumai-cho, Showa-ku, Nagoya, 466-8550 Japan

**Keywords:** Hirschsprung’s disease, Preoperative management, Irrigation, Single-stage surgery

## Abstract

**Background:**

Preoperative management of Hirschsprung’s disease (HD) is currently being conducted with the goal of performing single-stage radical surgery without ileostomy.

**Methods:**

We retrospectively reviewed HD cases between 2013 and 2022, as well as their outcomes related to preoperative management.

**Results:**

Thirty-nine patients with HD were included in this study, including short-segment HD (30 cases), long-segment HD (4 cases), and total colonic aganglionosis (5 cases). Among these 39 patients, 95% (37 of 39 patients) underwent single-stage radical surgery after management with glycerin enema use (n = 13), irrigation with tube insertion each time irrigation was performed (n = 13), and irrigation using a tube placed in the bowel (n = 11).

**Conclusions:**

Preoperative management of patients with HD allowed for single-stage surgery of long-segment HD and total colonic aganglionosis. Cases that could be managed without performing an emergency enterostomy during the neonatal period were managed with irrigation until radical surgery was performed.

## Background

Hirschsprung’s disease (HD) occurs in approximately 1 of every 7000 births, with male:female ratios of 4.1:1 among patients with short-segment HD and 2.4:1 among those with long-segment HD [1]. A lack of enteric ganglia in the hindgut could cause HD, resulting in abdominal distension symptoms. The classifications of HD include short-segment HD (aganglionosis up to the sigmoid colon–descending colon junction) [2] and long-segment HD (aganglionosis proximal to the sigmoid colon–descending colon junction but with ganglion cells present in some portions of the colon) [2]. These classifications also comprise total colonic aganglionosis (TCA; aganoglionosis of the entire colon and < 50 cm of the small bowel proximal to the ileocecal valve) [3] and extensive aganglionosis (aganglionosis extending to > 50 cm of the small bowel proximal to the ileocecal valve) [4]. Pull-through surgery has been adopted as a radical surgery for HD worldwide [5]. The outcomes of single-stage pull-through (SSPT) surgery for HD include lower readmission rates and lower rates of additional surgery compared with those associated with multi-stage surgery [6]. Additionally, performing SSPT surgery in the nonneonatal period could be more appropriate than that in the neonatal period to avoid postoperative perianal excoriation, anstomotic strictures, anastomotic leakage, postoperative enterocolitis, and incomplete continence in neonates [7]. Therefore, elective surgery might be selected for HD. However, appropriate decompression is essential to enable enteral feeding for patients with HD. Although rectal irrigation with saline using a soft rectal tube effectively decompresses bowel dilatation in these patients, its effect is usually limited in those with short-segment HD. Cases of long-segment HD, TCA, and extensive aganglionosis often require enterostomy. We use laparoscopic pull-through surgery when they are 3 to 4 months old and weigh at approximately 6 kg [8, 9]. To prevent functional bowel obstruction and enable appropriate body weight gain, preoperative bowel management was routinely performed before SSPT surgery for HD including TCA. We describe our preoperative bowel management for patients with HD.

## Methods

**Fig. 1:**
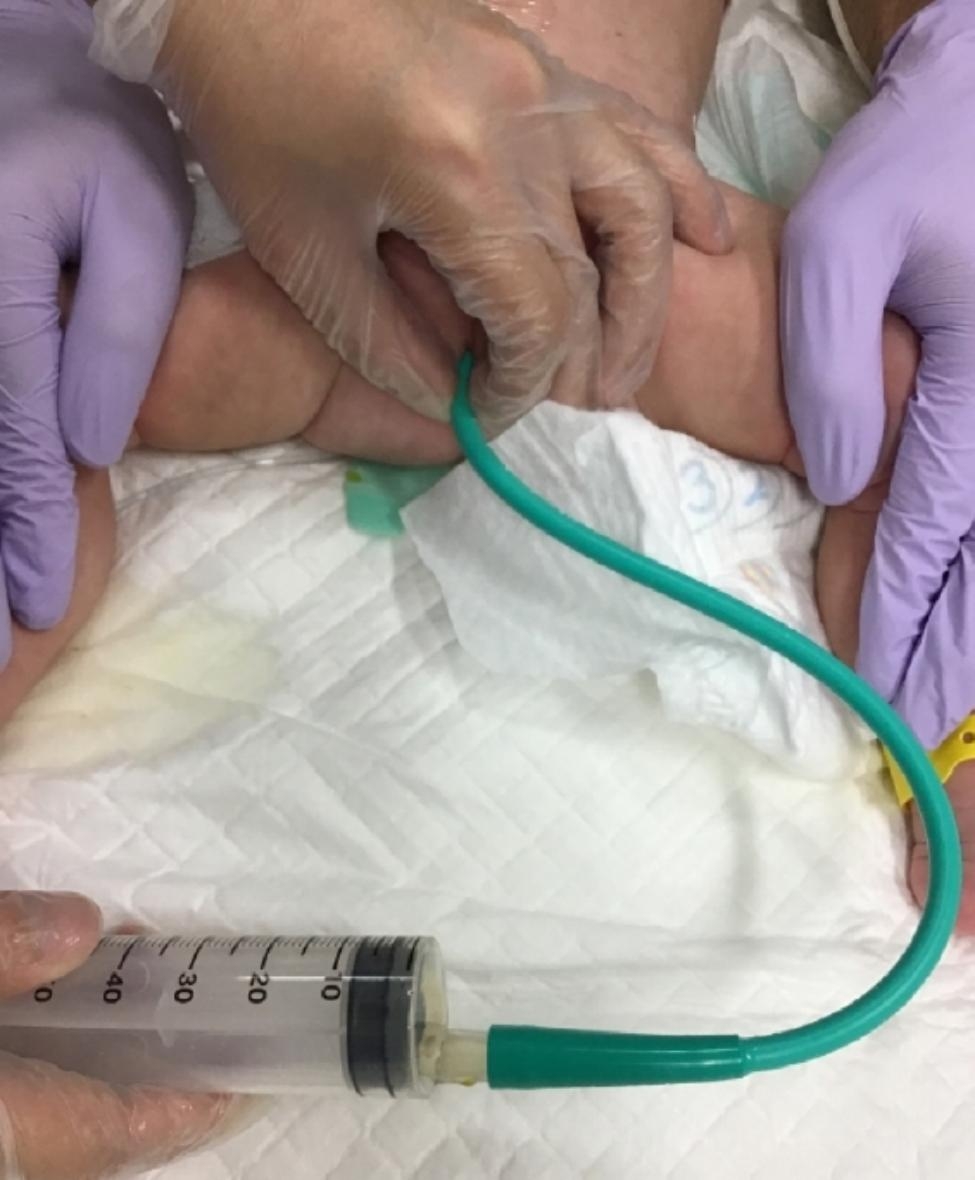
Home rectal irrigation using a Phycon tube. Home rectal irrigation is performed by inserting a 12-Fr trans-anal tube (Phycon tube)

**Fig. 2:**
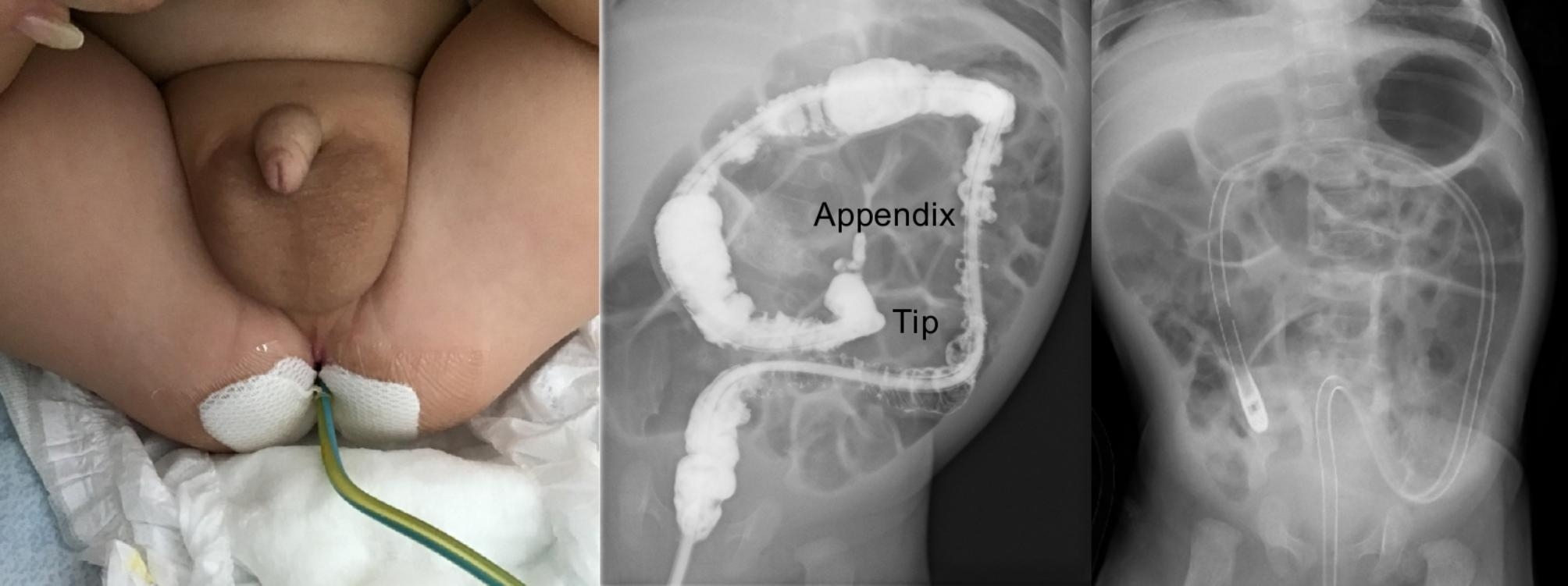
Home rectal irrigation using a trans-anal indwelling tube. Home rectal irrigation is performed using a 12-Fr trans-anal indwelling tube (enteral feeding tube). The tip is placed in the dilated bowel (usually the transverse or ascending colon) under fluoroscopy and the tube is firmly fixed to the buttocks

We retrospectively reviewed HD cases that were diagnosed between January 2013 and August 2022 at our institution. Patients who underwent emergent laparotomy due to perforation or suspected intestinal atresia were excluded from this study. All cases were diagnosed as HD based on the pathological evaluation of the rectal biopsy specimen or intraoperative biopsy specimen when cases required enterostomy. Our institution’s first choice of preoperative management of HD is glycerin enema (GE) use, followed by irrigation using a trans-anal indwelling tube for cases unresponsive to GE and enterostomy for cases unresponsive to irrigation. When short-segment HD cases were suspected based on the preoperative contrast study results and unresponsive to GE use, irrigation was performed by inserting a 12-Fr trans-anal tube (Phycon tube; Fuji Systems Corporation) for each irrigation (Fig. 1). For HD cases unresponsive to the aforementioned irrigation, irrigation was performed using a 12-Fr trans-anal indwelling tube (Enteral Feeding Tube; Kangaroo) whose tip was placed in the dilated bowel (usually the transverse or ascending colon). The indwelling tube was placed during a waking state under fluoroscopy and firmly fixed to the buttocks using tape and tube-supporting thread (Fig. 2). Tube insertion was usually performed within 1 to 2 min. Irrigation was performed one to three times per day by repeatedly injecting and recovering normal saline through the tube until it was confirmed that there was no abdominal distention or contaminated stool juice. This was repeated until radical surgery was performed. To minimize complications such as perforation, a sufficient saline volume for irrigation was determined using fluoroscopy and injected. Irrigation was performed for the patients at home by their parents. Their parents were taught how to perform irrigation using the tube during hopitalization, and the patients were discharged after their parents mastered the irrigation technique. When tube slippage occurred, or when the tube was accidentally removed or obstructed, the parents and the patient returned to our hospital so that it could be reinserted. When irrigation with an indwelling tube failed to control abdominal distension, enterostomy was performed.

Each single-stage radical surgery was performed with a single umbilical incision plus one port. Short-segment and long-segment HD cases underwent the laparoscopic Swenson procedure [8], whereas TCA and extensive aganglionosis cases underwent laparoscopic restorative proctocolectomy with an ileal-J-pouch anal canal anastomosis [9]. We chose to perform elective surgery for infants when they were 3 to 4 months old and weighed at approximately 6 kg because the laparoscopic Swenson and restorative proctocolectomy procedures require sufficient space for manipulation. Owing to the development of surgical techniques and instruments, minimally invasive surgery for infants weighing less than 5 kg has become feasible and safe [10, 11]; however, further technical advances are still required for these restricted spaces, and one study has shown that laparoscopic surgery for infants weighing less than 5 kg significantly increased perforation and nerve injury [12, 13]. Patients who were born prematurely or had a low birth weight did not necessarily grow enough to allow a simple surgical procedure at 3 to 4 months old; therefore, we chose to perform elective surgery when the patient reached a weight of approximately 6 kg instead.

We evaluated whether preoperative management of patients with HD would allow for SSPT surgery. Patients’ background, preoperative management methods, and several HD-associated enterocolitis (HAEC) were also evaluated. The type of HD was determined based on histological data of the excised segment.

### Ethical approval

This study was performed in accordance with the ethical standards of the 1964 Declaration of Helsinki and its later amendments or comparable ethical standards. This was a retrospective study. Patients were not required to provide informed consent to participate in the study because the analysis used anonymous clinical data obtained after each patient agreed to treatment by written informed consent. We applied the opt-out method to obtain consent for this study using a poster approved by the Institutional Review Board of Nagoya University Graduate School of Medicine. This study was approved by the Institutional Review Board of our institution (2022 − 0207).

## Results

**Fig. 3:**
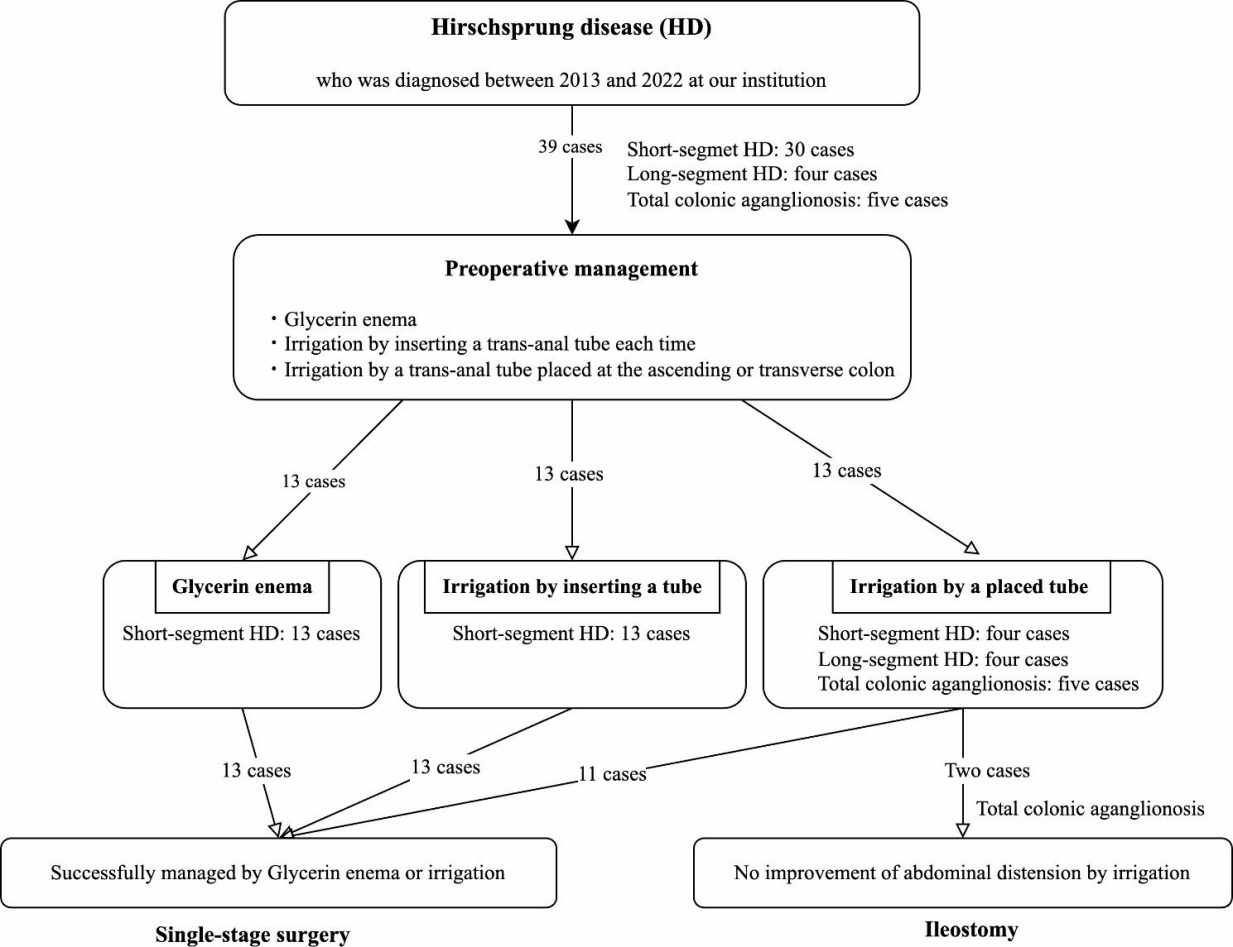
Flowchart of this study

The flowchart of this study is presented in Fig. 3.

### Patients’ background

There were 39 patients with HD in this study (Table 1). The types of HD included short-segment HD (30 cases), long-segment HD (four cases), and TCA (five cases). Additionally, 82% (32 of 39 patients) of the patients were male. Comorbidities included Trisomy 21 (seven cases) and Mowat-Wilson syndrome (one case). The median gestational age was 38 weeks 6 days (interquartile range, 37 weeks 6 days–39 weeks 5 days). The median body weight was 2948 g (interquartile range, 2744–3261 g). Preoperative HAEC occurred in four patients (10%). Radical surgery was performed at median age of 4 months (range, 2.5–5.5 months). The median weight at the time of surgery was 6.2 kg (range, 5.6–7.4 kg). For patients with Trisomy 21, the median follow-up period was 20 months (range, 0–105 months). One of these patients required irrigation management; good stool control was achieved in the other patients.

**Table 1:** Patients’ demographics in this study

Patients’ background of 39 HD cases	
Gestational age, median (interquartile range)	38w6d (37w6d–39w5d)
Male, n (%)	32 (82%)
Type of HD based on histological data of the excised segment	
Short-segment HD, n (%)	30 (77%)
Long-segment HD, n (%)	Four (10%)
Total colonic aganglionosis, n (%)	Five (13%)
≤ 5 cm small bowel proximal to the ileocecal valve > 5 cm and < 50 cm small bowel proximal to the ileocecal valve	Three cases Two cases
Comorbidities	
21 Trisomy, n (%)	Seven (18%)
Mowat-Wilson syndrome, n (%)	One (3%)

HD, Hirschsprung’s disease

### Preoperative management by GE use

Thirteen short-segment HD cases were successfully managed by GE use (Table 2). Long-segment HD and TCA could not be managed by GE use and required irrigation. Preoperative HAEC occurred in 2 of 13 patients (15%). However, all 13 patients underwent SSPT surgery without irrigation or enterostomy.

### Irrigation by inserting a trans-anal indwelling tube each time

Thirteen short-segment HD cases were successfully managed using irrigation with an indwelling tube that was inserted each time irrigation was performed (Table 2). Long-segment HD and TCA cases could not be managed by this type of irrigation and required trans-anal indwelling tube placement or enterostomy. Preoperative HAEC occurred in 1 of 13 patients (8%). However, all 13 patients underwent SSPT surgery without indwelling tube placement or enterostomy.

**Table 2:** Details of each bowel management and result

	Glycerin enema (n = 13)	Irrigation by inserting a tube (n = 13)	Irrigation by a placed tube (n = 13)
**Patients’ background**			
Type of HD			
Short-segment HD, n (%)	13 (100%)	13 (100%)	Four (31%)
Long-segment HD, n (%)	–	–	Four (31%)
Total colonic aganglionosis, n (%)	–	–	Five (38%)
**Management outcome**			
Tube insertion period ^†^	–	–	3 (1.5–4) months
Complications			
Tube trouble (accidental removal and obstruction), n	–	–	12 times in five patients
Preoperative HAEC incidence, n (%)	Two (15%)	One (8%)	Three (23%)
Age at radical surgery, median ^†^	5 (2–12) months	3 (3–4) months	4 (3–5) months
Wt at radical surgery, median ^†^	6.2 (5.6–7.4) kg	6.6 (5.7–7.5) kg	6.3 (5.7–7.5) kg
Single-stage surgery, n (%)	13 (100%)	13 (100%)	11 (85%)*

HD, Hirschsprung’s disease

HAEC, Hirschsprung’s disease associated enterocolitis

Wt, body weight

*: Two total colonic aganglionosis cases required ileostomy due to sustanined abdominal distension even using irrigation

†: interquartile range

### Irrigation by a trans-anal indwelling tube placed in the bowel

Thirteen cases were managed using irrigation by a trans-anal indwelling tube placed in the bowel (Table 2). Eleven cases were successfully managed by this method; however, the other two cases were unresponsive to irrigation and required ileostomy. The types of HD successfully managed by irrigation through an indwelling tube placed in the bowel included short-segment HD (four cases), long-segment HD (four cases), and TCA (three cases). However, two patients with TCA experienced sustained abdominal distension even with irrigation and required ileostomy. Preoperative HAEC occurred in 3 of the 13 patients (23%). The median tube insertion period was 3 months. During the preoperative management period, tube problems, accidental tube removal, and obstruction occurred 12 times in five patients and required presentation to the hospital to exchange trans-anal indwelling tubes. There were no other complications such as perforation or dermatitis. SSPT surgery was performed for 85% of patients (11 of 13 patients).

## Discussion

During this study, we demonstrated that 95% (37 of 39 patients) of patients with HD successfully underwent SSPT surgery following our preoperative management of HD. Cases that could be managed without performing emergent enterostomy during the neonatal period were managed by irrigation until radical surgery was performed. Although some cases of TCA required enterostomy, parents could easily perform preoperative management of patients with HD, allowing for SSPT surgery for cases including long-segment HD and TCA; hence, this method should be selected first for HD cases.

Pull-through surgery is the standard surgical method for HD cases, although there is no consensus regarding the superior HD treatment method. However, we adopted the single-stage laparoscopic trans-anal pull-through modified Swenson procedure for short-segment and long-segment HD [8] and laparoscopic restorative proctocolectomy with an ileal-J-pouch anal canal anastomosis for TCA and extensive aganglionosis [9]. No specific advantages of radical surgery during the immediate neonatal period have been demonstrated [7, 10, 14]. Furthermore, radical surgery is usually performed electively within 2 to 3 months if the condition of the infant is stable and the bowel is decompressed [12, 15]. SSPT surgery is associated with significantly lower readmission and re-operation rates than multi-stage pull-through surgery [6]; furthermore, the patients in the multi-stage surgery group had worse conditions. The presence of an ileostomy for a long period of time could cause complications such as high output of the stoma and peristomal skin excoriation [14, 16]. The European Paediatric Surgeons’ Association Survey reported that 67% of the members performed delayed pull-through surgery [15, 17]. Regarding delayed pull-through surgery, preoperative management was essential to prevent HAEC and decompress bowel distension until the time of radical surgery.

For short-segment HD, GE use was first selected at our institution owing to its ease of use. A previous study showed that GE use only relieved obstruction in approximately 80% of HD cases [16, 18]. However, only 43% (13 of 30 cases) of short-segment HD cases were successfully managed by GE use only during this study, and 57% (17 of 30 cases) of short-segment HD cases required irrigation by an indwelling tube. Rectal irrigation at home was feasible and effective for HD cases, allowed delayed pull-through surgery [17, 19], and contributed to decreasing perianal excoriation, anastomotic site strictures and leakage, HAEC, and incomplete continence [7]. We also adopted home rectal irrigation via the insertion of an indwelling tube each time irrigation was performed as previously described because it had been reported that rectal irrigation could effectively decompress the bowel in approximately 75% of HD cases [10, 14]. However, long-segment HD and further extended agangolionosis cases were not suitable for rectal irrigation [17, 19] because an indwelling tube could not be effective unless the tip of the tube was placed in the dilated bowel. Only short-segment HD cases were successfully managed using irrigation with an indwelling tube inserted each time irrigation was performed during this study. Moreover, some short-segment HD cases additionally required irrigation using an indwelling tube. If an indwelling tube was deeply inserted in the dilated bowel, then this irrigation technique could contribute to bowel decompression. However, blinded deep insertion and irrigation could cause perforation [18] and are very dangerous procedures.

The novel point of this study is that preoperative management allowed for SSPT surgery, even for TCA cases. The descriptions of our preoperative management protocol and fixation of the tube could aid in their application by other institutions. Although rectal irrigation at home was feasible and effective for patients with short-segment HD, it was not suitable for patients with long-segment HD or further extedend aganglionosis. Bowel distension that could not be decompressed by rectal irrigation required enterostomy. Enterostomy could be complicated by electrolyte disturbances, stoma prolapse, or peristomal skin excoriation [19]. However, we consider enterostomy too invasive for managing long-segment HD. Indwelling tube irrigation effectively decompressed the dilated bowel of patients with HD, including long-segment HD and TCA. During this study, 100% (four of four patients) of long-segment HD patients and 60% (three of five patients) of TCA patients who underwent tube placement for irrigation were successfully managed, thus allowing for SSPT surgery. Two cases of TCA were unresponsive to indwelling tube irrigation and required ileostomy. It was only natural that irrigation using an indwelling tube placed in the bowel was unable to effectively wash-out the terminal ileum. Hence, cases of aganglionosis extending to the terminal ileum were theoretically unresponsive to total colon irrigation.

The effect of irrigation on long-segment HD and TCA should be considered. All long-segment HD cases were successfully managed by irrigation, and preoperative HAEC occurred only in one out of four patients. Theoretically, tube irrigation decompressed bowel distension until the ascending colon. Unlike long-segment HD, whether the irrigation tube in the ascending colon can decompress bowel distention with TCA is questionable. During this study, three cases were successfully managed by irrigation, whereas the other two sustained abdominal distension despite irrigation and required ileostomy. Two cases of aganglionosis of the entire colon and 38 cm and 20 cm of the terminal ileum were unresponsive to irrigation, suggesting that the irrigation tube in the ascending colon was unable to decompress the terminal ileum more than 20 cm from ileocecal valve. Three cases of aganglionosis of the entire colon and ≤ 5 cm of the terminal ileum was successfully managed by irrigation, meaning that aganglionosis until approximately 5 cm of the terminal ileum could be managed by irrigation. However, slight distension of the abdomen continued in these cases before surgery, and parents appropriately performed irrigation repeatedly when they noticed abdominal distention. Although preoperative HAEC only occurred in one patient, successful irrigation management for TCA depends on the aganglionic distance from the ileocecal valve and the parents’ efforts; therefore, irrigation management is not necessarily effective for all TCA cases.

## Conclusions

After irrigation, 95% (37 of 39 patients) of patients with HD, including long-segment HD and TCA, successfully underwent SSPT surgery. Cases that could be managed without performing emergent enterostomy during the neonatal period were managed by irrigation until radical surgery was performed. Irrigation with an indwelling tube in the ascending or transverse colon allowed for SSPT surgery of cases of aganoglionosis of the entire colon and approximately 5 cm of the terminal ileum.

## Data Availability

The datasets used and/or analyzed during the current study are available from the corresponding author on reasonable request.

## Abbreviations

HD: Hirschsprung’s disease
GE: Glycerin enema
HAEC: Hirschsprung’s disease-associated enterocolitis
TCA: total colonic aganglionosis
SSPT: single-stage pull-through

## References

[1] Russell MB, Russell CA, Niebuhr E (1994). An epidemiological study of Hirschsprung’s Disease and additional anomalies. Acta Paediatr.

[2] Kawaguchi AL, Guner YS, Sømme S, Quesenberry AC, Arthur LG, Sola JE (2021). Management and outcomes for long-segment Hirschsprung Disease: a systematic review from the APSA Outcomes and Evidence Based Practice Committee. J Pediatr Surg.

[3] Hoehner JC, Ein SH, Shandling B, Kim PCW (1998). Long-term morbidity in total colonic aganglionosis. J Pediatr Surg.

[4] Moore SW (2015). Total colonic aganglionosis and Hirschsprung’s Disease: a review. Pediatr Surg Int.

[5] De la Torre-Mondragón L, Ortega-Salgado JA (1998). Transanal Endorectal pull-through for Hirschsprung’s Disease. J Pediatr Surg.

[6] Sulkowski JP, Cooper JN, Congeni A, Pearson EG, Nwomeh BC, Doolin EJ (2014). Single-stage versus multi-stage pull-through for Hirschsprung’s Disease: practice trends and outcomes in infants. J Pediatr Surg.

[7] Lu C, Hou G, Liu C, Geng Q, Xu X, Zhang J (2017). Single-stage transanal endorectal pull-through procedure for correction of Hirschsprung Disease in neonates and nonneonates: a multicenter study. J Pediatr Surg.

[8] Yokota K, Uchida H, Tainaka T, Tanaka Y, Shirota C, Hinoki A (2018). Single-stage laparoscopic transanal pull-through modified Swenson procedure without leaving a muscular cuff for short- and long-type Hirschsprung Disease: a comparative study. Pediatr Surg Int.

[9] Nakagawa Y, Yokota K, Uchida H, Hinoki A, Shirota C, Tainaka T (2022). Laparoscopic restorative proctocolectomy with ileal-J-pouch anal canal anastomosis without diverting ileostomy for total colonic and extensive aganglionosis is safe and feasible with combined Lugol’s iodine staining technique and indocyanine green fluorescence angiography. Front Pediatr.

[10] Kyrklund K, Sloots CEJ, de Blaauw I, Bjørnland K, Rolle U, Cavalieri D (2020). ERNICA guidelines for the management of rectosigmoid Hirschsprung’s Disease. Orphanet J Rare Dis.

[11] Ponsky TA, Rothenberg SS. Minimally invasive surgery in infants less than 5 kg: experience of 649 cases. Surg Endosc. 2008;22(10):2214–9. 10.1007/s00464-008-0025-7. 10.1007/s00464-008-0025-7 18649102

[12] Bradnock TJ, Walker GM (2011). Evolution in the management of Hirschsprung’s Disease in the UK and Ireland: a national survey of practice revisited. Ann R Coll Surg Engl.

[13] Iwanaka T, Uchida H, Kawashima H, Nishi A, Kudou S, Satake R. Complications of laparoscopic surgery in neonates and small infants. J Pediatr Surg. 2004;39(12):1838–41. 10.1016/j.jpedsurg.2004.08.011. 10.1016/j.jpedsurg.2004.08.011 15616945

[14] Vriesman MH, Noor N, Koppen IJ, Di Lorenzo C, de Jong JR, Benninga MA (2020). Outcomes after enterostomies in children with and without motility disorders: a description and comparison of postoperative Complications. J Pediatr Surg.

[15] Zani A, Eaton S, Morini F, Puri P, Rintala R, van Heurn E (2017). European Paediatric Surgeons’ Association Survey on the management of Hirschsprung Disease. Eur J Pediatr Surg.

[16] Carcassonne M, Guys JM, Morrison-Lacombe G, Kreitmann B (1989). Management of Hirschsprung’s Disease: curative Surgery before 3 months of age. J Pediatr Surg.

[17] Lu C, Xie H, Li H, Geng Q, Chen H, Mo X (2019). Feasibility and efficacy of home rectal irrigation in neonates and early infancy with Hirschsprung Disease. Pediatr Surg Int.

[18] Gayer G, Zissin R, Apter S, Oscadchy A, Hertz M (2002). Perforations of the rectosigmoid colon induced by cleansing enema: CT findings in 14 patients. Abdom Imaging.

[19] Moore SW (2012). Total colonic aganglionosis in Hirschsprung Disease. Semin Pediatr Surg.

